
JOURNAL INFORMATION
==============================
NLM Title Abbreviation: Gastroenterol Rep (Oxf)
Journal ID: Gastroenterol Rep (Oxf)
NLM Journal ID: 101620508
Journal ID: gastro

Gastroenterology Report

EISSN: 2052-0034
Publisher: Oxford University Press

ARTICLE INFORMATION
==============================
PMCID: PMC6454837
DOI: 10.1093/gastro/goz013
Article ID: goz013
Article version: 1
Subjects: Chinese Abstracts

Chinese Abstract – Volume 7 Issue 2*

This article abstract was originally published in English. This translation has not been verified and should not be relied upon—it is provided for reference purposes only. The Publishers have not checked this translation and accept no liability for completeness or accuracy of this translation or the use of this translation for whatever purpose. This translation may be incomplete and inaccurate in whole or in part. If you need to rely upon a translation of this abstract, a professional human translator should be engaged to supply an accurate translation of the original English. When referencing articles from this journal, please always refer to the original English version, rather than a translated equivalent.

Print publication date: 2019 Apr
Electronic publication date: 2019 Apr 9
Volume: 7
Issue: 2
First page: C1
Last page: C2
Copyright: © The Author(s) 2019. Published by Oxford University Press and Sixth Affiliated Hospital of Sun Yat-Sen University
Copyright year: 2019
License: This is an Open Access article distributed under the terms of the Creative Commons Attribution License (http://creativecommons.org/licenses/by/4.0/), which permits unrestricted reuse, distribution, and reproduction in any medium, provided the original work is properly cited.
License URL: https://creativecommons.org/licenses/by/4.0/

Page count: 2

==============================
OXFORD

*Gastroenterology Report*, 7(2), 2019, 1–2

doi: 10.1093/gastro/goz013
Abstracts

ABSTRACTS

### Chinese Abstract – Volume 7 Issue 2*

### Review （综述）

### Surgery in the age of biologics
### 生物治疗时代的外科手术

自20世纪90年代后期首个抗肿瘤坏死因子抗体问世后，生物治疗为炎症性肠病（IBD）患者的药物治疗带来了革命性的改变。然而，外科手术在IBD的治疗中仍然占据重要作用。无论是对于克罗恩病的小肠切除术还是对溃疡性结肠炎的结肠切除术， 手术切除率长期以来一直在下降但降幅并不显著。不断增多的生物制剂可越来越多地满足不同情况下IBD患者的治疗需求。于是， 出现了一系列关于生物制剂如何影响手术需求及IBD病程的问题。考虑到生物制剂越来越早且越来越多地应用于IBD患者，了解这些药物对手术决策和外科预后的影响，对于结直肠外科医生和胃肠科医生的临床实践是必需的。本文总结了生物治疗与IBD手术方面的相关文献，重点是探讨在“手术治疗时代”，生物制剂对手术频率、类型及并发症的影响。

### Additional gastrectomy in early-stage gastric cancer after noncurative endoscopic resection: a meta-analysis
### 早期胃癌非根治性内镜切除术后追加胃切除术：meta 分析

背景：对于行非根治性内镜切除术的早期胃癌患者，追加胃切除术的作用尚不明确。本研究旨在探讨早期胃癌患者非根治性内镜切除术的危险因素，并评价追加胃切除术的疗效。

方法：在MedLine、Web of Science和EMBASE数据库中检索有关接受非根治性内镜切除术后追加胃切除术的文献报道。首先分析非根治性内镜切除术后肿瘤残留及淋巴结转移的危险因素， 然后评估追加胃切除术患者的生存结果，包括5年总生存率、5年无病生存率和5年疾病特异生存率。

结果：本研究纳入了21项研究共4870例病例。分析发现，肿瘤残留与肿瘤较大（>3 cm）（OR = 2.81, P < 0.001）、肿瘤为未分化类型（OR = 1.78, P = 0.011）以及切缘阳性（OR = 9.78, P < 0.001）有关。淋巴结转移与肿瘤较大（>3 cm）（OR = 1.73, P < 0.001）、肿瘤大体分型提高（OR = 1.60, P = 0.035）以及肿瘤浸润较深（OR = 2.68, P < 0.001）有关。追加胃切除术的患者较未追加者5年总生存率（HR = 0.34, P < 0.001）、5年无病生存率（HR = 0.52, P = 0.001）和5年疾病特异生存率（HR = 0.50, P < 0.001）均显著提高。而且老年患者也能从追加胃切除术中取得总生存获益（HR = 0.41, P = 0.001）。

结论：追加胃切除术及淋巴结清扫可以延长早期胃癌患者非根治性内镜切除术后生存期。然后， 应对这些患者进行危险分层以选择适宜的病例追加胃切除术，以避免过度治疗。

### Original article （论著）

### Radiation-induced injury on surgical margins: a clue to anastomotic leakage after rectal-cancer resection with neoadjuvant chemoradiotherapy?
### 新辅助放化疗加直肠癌切除术后外科切缘的放射性损伤：吻合口漏的预判线索

背景：对于直肠癌切除术后吻合口状态及其对吻合口漏的影响，目前鲜有研究报道。本研究旨在分析新辅助放化疗+直肠前切除术后外科切缘可能受到的放射性损伤，以及其与吻合口漏发生的关系。

方法：回顾性纳入2014－2015年间连续收治的161例直肠前切除手术患者， 包括行新辅助放化疗、单纯新辅助化疗或未行术前新辅助治疗者。采用一种专门的组织病理学评分和黏膜下层微血管密度， 来对手术切缘标本进行评估。采用倾向评分匹配方法来平衡基线资料。分析吻合口漏与组织病理学特征的相关性。

结果：54例新辅助放化疗患者中13例术后发生吻合口漏，48例新辅助化疗患者中5例术后发生吻合口漏， 而59例未行新辅助治疗的患者中有7例出现术后吻合口漏。倾向评分匹配后， 新辅助放化疗、新辅助化疗和未行新辅助治疗三组患者近切缘组织病理评分中位数（范围）分别为3（0－8）、0（0－3）和0（0－2）（P < 0.001），远切缘组织病理评分分别为4（2－9）、0（0－4）和0（0－3）（P < 0.001）；近切缘微血管密度平均数（标准差）分别为21.7（7.9）、27.2（8.6）和27.3（9.4）（P = 0.003）， 远切缘微血管密度分别为18.1（9.3）、25.2（12.9）和24.9（7.4）（P < 0.001）。在新辅助放化疗患者中，吻合口漏的发生与组织病理学评分增高（P = 0.003）及微血管密度减少（P = 0.004）密切相关。

结论：新辅助放化疗后，直肠癌切除术的外科切缘存在一定程度的放射性损伤。近切缘放射性损伤越严重，吻合口漏发生概率越高。

*This article abstract was originally published in English. This translation has not been verified and should not be relied upon—it is provided for reference purposes only. The Publishers have not checked this translation and accept no liability for completeness or accuracy of this translation or the use of this translation for whatever purpose. This translation may be incomplete and inaccurate in whole or in part. If you need to rely upon a translation of this abstract, a professional human translator should be engaged to supply an accurate translation of the original English. When referencing articles from this journal, please always refer to the original English version, rather than a translated equivalent.
本文摘要最初是以英文发表。中文摘译未获认证，谨供参考。出版社没有对中文摘译进行核验，不负责译文的完整性和准确性，也不会将其用以其他目的。中文摘译可能会出现部分不完整或不准确的情况。如果你需要准确获悉摘要，最好能请一位专业译者针对英文原文进行翻译。阅读杂志文章，请参考英文原文，不推荐中文翻译。

### Pre-operative total parenteral nutrition improves post-operative outcomes in a subset of Crohn's disease patients undergoing major abdominal surgery

术前全胃肠外营养可改善行主要腹部手术的克罗恩病患者的手术疗效

背景：尽管克罗恩病（CD）的药物治疗取得了重大进展，但仍有相当一部分患者在诊断后5年内需要接受外科手术治疗。营养不良是胃肠手术疗效欠佳的独立危险因素。术前全胃肠外营养（TPN）在CD患者中的应用价值，各项研究数据并不一致，而且缺少生物治疗时代的相关研究数据。本研究旨在明确TPN在CD手术患者中的应用价值。

方法：这是一项在一家三级转诊中心完成的回顾性队例研究。纳入接受主要腹部手术的CD患者， 按术前有无接受TPN治疗分为TPN组与非TPN组。比较两组患者术后30天感染性与非感染性并发症发生率。

结果：纳入2007年3月至2017年3月间行主要腹部手术的144例CD患者，其中55例接受术前TPN治疗，非TPN组89例。21例（14.6%）患者术后出现感染性并发症， 其中TPN组与非TPN组发生率分别为18.2%和12.3%（$P = 0.34$）；23例（15.9%）术后发生非感染性并发症， 其中TPN组与非TPN组发生率分别为14.5%和16.9%（$P = 0.71$）。采用多因素分析校正两组间基线疾病严重度和营养不良程度的差异后，术前TPN治疗 ≥60天的患者，其术后发生非感染性并发症的风险显著降低（OR = 0.07，95% CI：0.01–0.80，$P = 0.03$）。近6个月内体重下降 >10% 则是术后并发症的危险因素。

结论：对于营养不良的CD患者，TPN是安全的，而且可以使其获得与营养正常CD患者相当的手术疗效。术前行≥60天TPN治疗可减少术后非感染性并发症的发生，而且并不会增加感染性并发症的发生概率。

### Early colonoscopy in patients with acute diverticular bleeding is associated with improvement in healthcare-resource utilization

对急性憩室出血患者进行早期结肠镜诊治可提高医疗资源利用率

背景：在发达国家，憩室出血是严重的急性下消化道出血最常见的原因。早期结肠镜（≤24小时）的作用仍然存在争议， 目前尚无早期肠镜应用于急性憩室出血的数据。本研究旨在通过美国全国住院患者样本库，评估结肠镜诊治的时机对急性憩室出血患者预后的影响。

方法：采用第9版国际疾病分类（ICD 9），对全国住院患者样本库中2012–2014年间的病例进行选择并提取资料。纳入出院诊断（包括主要诊断或第二诊断）为憩室病合并出血和憩室炎合并出血的病例， 排除年龄不满18周岁以及住院期间未行结肠镜检查的病例。首要结局指标为住院时间和住院总费用。

结果：共计88,600例患者纳入研究， 其中45,020例（50.8%）在入院24小时内接受了结肠镜检查（早期肠镜组），而另外43,580例（49.2%）则是在入院24小时后行结肠镜检查（晚期肠镜组）。早期肠镜组较晚期肠镜组患者住院时间显著缩短（3.7天 vs 5.6天，$P < 0.001$），住院总花费显著降低（$9317 vs $11,767，$P < 0.001$）。两组患者病死率的差异无统计学意义（0.7% vs 0.8%）。对潜在致混淆因素进行校正后，两组间住院时间和住院总费用的差异仍然具有统计学意义。

结论：对于急性憩室出血，早期结肠镜诊治可显著缩短住院时间，降低住院费用， 而且并不会增加病死率。选择适宜的急性憩室出血病例进行早期结肠镜诊治， 具有节省医疗资源的潜在意义。进一步研究需明确哪些憩室出血患者能从早期结肠镜诊治中获益。

### Vedolizumab for chronic antibiotic-refractory pouchitis

维多珠单抗治疗慢性抗生素难治性储袋炎

背景：慢性抗生素难治性储袋炎（CARP）是回肠储袋–肛管吻合术（IPAA）的一个并发症，治疗上存在巨大挑战。维多珠单抗是一种肠道选择性的抗α4β7整合素单克隆抗体，现已应用于CARP患者，但其疗效数据报道不多。本研究旨在评估维多珠单抗作为诱导治疗，应用于CARP患者的有效性和安全性。

方法：从一个单中心的历史队列中， 筛选出2015年1月至2017年6月间接受维多珠单抗治疗的CARP患者。病例纳入标准：改良储袋疾病严重指数（mPDAI）评分≥5的急性储袋炎；但如果没有mPDAI评分则以临床医生作出急性储袋炎的诊断为准。于治疗前及治疗后3个月，通过储袋镜和门诊检查来进行mPDAI评分。

结果：19例患者纳入研究，平均年龄（26.7±12.8）岁，其中男性10例（53%）。9例（47%）在结肠切除术前曾接受抗肿瘤坏死因子（TNF）药物治疗， 10例（53%）患者在IPAA术后接受过抗TNF药物治疗。维多珠单抗治疗后， 6例（32%）患者mPDAI症状评分显著改善（$P = 0.031$）；14例（74%）患者mPDAI内镜评分和总评分均显著改善，中位下降均为2分（$P = 0.031$）。仅2例（11%）患者出现药物不良反应，另有4例（21%）患者需针对CARP行再次手术治疗。

结论：本研究显示， 维多珠单抗治疗CARP患者安全有效。而这些结果尚需更大样本量的研究证实。

### Bone marrow-derived CXCR4-overexpressing MSCs display increased homing to intestine and ameliorate colitis-associated tumorigenesis in mice

过表达CXCR4的骨髓间充质干细胞通过增强的肠道归巢能力抑制小鼠肠炎相关肿瘤的形成

背景：骨髓来源间充质干细胞（MSC）用于炎症性肠病（IBD）及IBD相关肠癌的治疗潜力已受到越来越多的关注。然而，MSC究竟是抵制还是促进肿瘤形成仍然存在争议。基质细胞衍生因子1（SDF-1）/ C-X-C趋化因子受体4（CXCR4）在MSC归巢过程具有非常关键的作用。本研究旨在评估过表达CXCR4的MSC在IBD瘤变过程中的作用。

方法：应用携带CXCR4或绿色荧光蛋白（GFP）的慢病毒载体转染MSC。通过细胞趋化实验和侵袭实验检测CXCR4表达情况。采用偶氮甲烷和葡聚糖硫酸钠（DSS）制作小鼠结肠炎成瘤模型。将实验小鼠分为3组，分别注射磷酸盐缓冲液（PBS）、MSC-GFP或MSC-CXCR4。

结果：与MSC-GFP组小鼠相比， MSC-CXCR4小鼠体重下降程度减少，结肠相对更长，肿瘤数量更少，肿瘤负荷减轻；此外，结肠组织中促炎细胞因子表达和STAT3磷酸化水平均下调。

结论：过表达CXCR4的MSC表现出抑瘤功能， 其机制可能是CXCR4促进MSC向炎性肠组织的归巢。

### Case report （病例报告）

### Retrieval of dislodged AmplifEYE using balloon dilator during colonoscopy

使用球囊扩张器取出结肠镜检查过程中掉落的 AmpliEYE

### Embolization of blood-supply artery followed by surgery for treatment of mesorectal Castleman's disease: case report and literature review

外科手术加动脉栓塞治疗直肠 Castleman 病：病例报告和文献复习

### Study protocol（研究方案）

### Design and rationale of a randomized–controlled trial of home-delivered meals for the management of symptomatic ascites: the SALTYFOOD trial

送餐入户方案治疗有症状腹水的前瞻性对照研究的设计与合理性：SALTYFOOD 试验
